# Widely Tunable Photonic Filter Based on Equivalent Chirped Four-Phase-Shifted Sampled Bragg Gratings

**DOI:** 10.1021/acsphotonics.4c01899

**Published:** 2025-01-27

**Authors:** Simeng Zhu, Bocheng Yuan, Mohanad Al-Rubaiee, Yiming Sun, Yizhe Fan, Ahmet Seckin Hezarfen, Stephen J. Sweeney, John H. Marsh, Lianping Hou

**Affiliations:** James Watt School of Engineering, University of Glasgow, Glasgow G12 8QQ, U.K.

**Keywords:** sampled Bragg grating, photonic filter, tunable filter, micro heater, optical frequency division, equivalent reconstruction technology

## Abstract

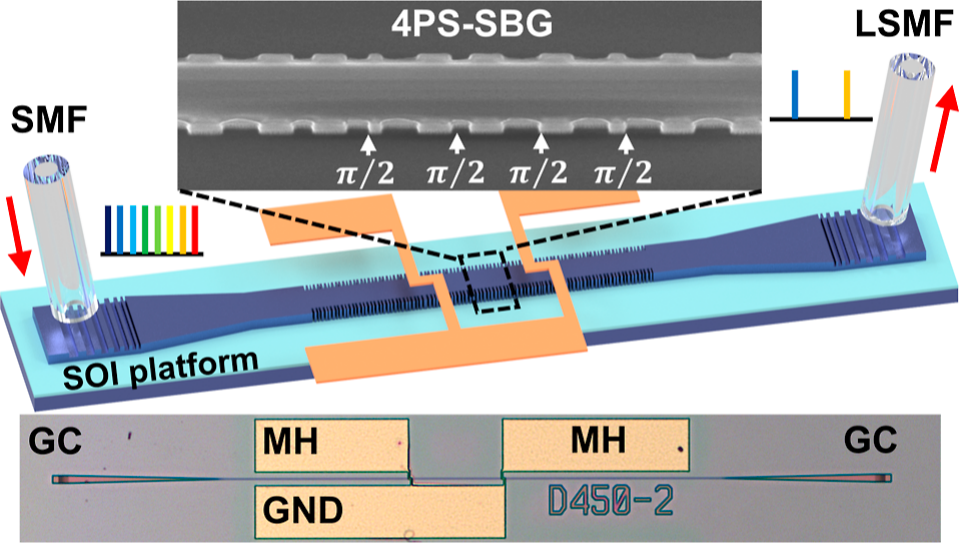

We have developed an integrated dual-band photonic filter (PF) utilizing equivalent chirped four-phase-shifted sidewall-sampled Bragg gratings (4PS-SBG) on a silicon-on-insulator platform. Using the reconstruction equivalent-chirp technique, we designed linearly chirped 4PS Bragg gratings with two π-phase shifts (π-PSs) positioned at 1/3 and 2/3 of the grating cavity, introducing two passbands in the + first order channel. Leveraging the significant thermo-optic effect of silicon, dual-band tuning is achieved through integrated microheaters (MHs) on the chip surface. By varying the injection currents from 0 to 85 mA into the MHs, the device demonstrates continuous and wide-range optical frequency division performance, with the frequency interval between the two passbands adjustable from 52.1 to 439.5 GHz. Four notable frequency division setups at 100, 200, 300, and 400 GHz were demonstrated using a 100 GHz, 1535 nm semiconductor passive mode-locked laser as the light source.

## Introduction

The increasing demand for higher bandwidth is positioning photonic systems as prime candidates for future telecommunications and radar technologies.^1^ Integrated photonic systems offer ultrawideband performance within compact dimensions and can naturally interface with fiber optic networks for signal transmission.^2−4^ To address the evolving operational demands of wavelength-division multiplexing (WDM) systems, data center optical networks, and microwave photonics, there is a pressing need for the development of tunable and reconfigurable multiband photonic filters (PFs).^5^ Multichannel PFs can effectively suppress unwanted signals in congested radio frequency (RF) spectrum environments while preserving multiple useful signals.^2,6^ As RF systems advance to operating frequencies above 30 GHz, into the millimeter-wave band, these filters must also provide wideband frequency tunability.^3^ Furthermore, they need to be compact, lightweight, and energy-efficient to meet the stringent requirements of future RF applications.^7,8^

PFs utilize the broadband tunability and reconfigurability of optical components to overcome the frequency tuning limitations that have constrained traditional RF filtering technologies over the past 20 years.^9−12^ Recent research has reported various designs for multifrequency PFs, which can be realized by cascading multiple single-channel PFs. For instance, this can be accomplishes by employing two microring resonator (MRR)-loaded Mach–Zehnder interferometers (MZIs)^13^ to achieve complex rectangular spectral responses, with a 100 GHz free spectral range (FSR) limitation, or two cascaded distributed feedback Bragg grating resonators (DFBRs)^14^ with a single cavity length of 800 μm.^15^ Another straightforward approach to achieving single-channel filtering is through the nonlinear effect of stimulated Brillouin scattering (SBS).^16^ In tunable filters based on silicon photonics, SBS is favored for its high selectivity and narrow bandwidth,^17,18^ but it also suffers from a narrow modulation range^18,19^ and large on-chip area requirements.^18−21^

Another approach to achieving a dual-frequency PF is to directly implement multiple passbands using a single PF. Examples include equivalent phase shift fiber Bragg gratings (EPS-FBG),^5^ based on phase-modulation-to-intensity-modulation (PM-IM) conversion, which feature a cavity length of 32 cm, a −3 dB bandwidth of 167 MHz, and a maximum tuning range of just 7.4 GHz. Similarly, the MRR offers a −3 dB bandwidth of 15 GHz and a FSR of 282 GHz^22^ but does not allow for independent tuning of each passband. And the EO-modulated phase shift Bragg grating (PS-BG)^23^ has a 14 pm line width dual channel with a modulation range of 113.4 GHz, but its passbands are also not independently tunable.

In this study, we designed and fabricated a tunable dual-passband PF on an SOI platform, enabling independent tuning of two passbands on a single grating waveguide. This approach utilizes an equivalent chirped^24^ 4PS-SBG with two π-PSs and the thermo-optic interaction of two microheaters (MHs). The equivalent chirp of the grating spatially separates the photons of the two passbands, enabling independent tuning of the passband wavelengths. This equivalent chirp is achieved by linearly modulating the micron-scale sampling period, significantly reducing manufacturing complexity compared to modulating the nanometer-scale seed grating period.^25^ Two MHs are placed above the positions of the two PS points. By leveraging the thermo-optic effect to alter the phase amplitude of the two PSs, the positions of the passbands can be precisely adjusted.^26^ The integration of the 4PS-SBG and MH offers greater design flexibility compared to multi-MRR and SBS filters, enabling the development of narrowband, tunable, and customizable multipassband filters. The integrated PF demonstrated a frequency spacing tuning range of 387 GHz, achieving a −3 dB bandwidth of 16 GHz for a single passband. Additionally, by using a semiconductor mode-locked laser (SMLL) in optical frequency division (OFD) experiments, the PF suppressed interference signals within the stopband spectral range, achieving excellent frequency division performance from 100 to 400 GHz, with a maximum side-mode suppression ratio (SMSR) exceeding 10 dB.

Compared to conventional MZI and MRR-based filters, the proposed PF provides narrower passband frequencies and a wide tunable frequency range that is not constrained by the FSR.^2^ Additionally, our approach reduces the number of resonators needed to process multiple signals, offering a compact solution for photonic integrated circuits (PICs). The devices were fabricated on a standard SOI platform using established manufacturing techniques and waveguide geometries, facilitating wafer-level integration of other multichannel filter components on the same platform. For a detailed performance comparison of our PF with other similar studies, please refer to Supporting Information, Part A.

## Design and Methods

For the refractive index modulation of the sampled grating, it can be written as the following^27^
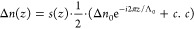
1where Δ*n*_0_ is the refractive index modulation amplitude of the seed grating, *z* is the position in the grating cavity, and Λ_0_ is the seed grating period. *s*(*z*) is the sampling function. The Fourier expansion of the *s*(*z*) can be expressed as follows
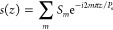
2where *m* represents the Fourier series, *S*_*m*_ denotes the corresponding channel intensity coefficient, and *P*_s_ refers to the sampling period. For the conventional sampled Bragg grating (C-SBG), each section, including the grating and nongrating parts, has a length equal to half of the sampling period *P*_s_. Within a complete sampling period *P*_s_, the corresponding *s*(*z*) for the C-SBG is given as follows^28^
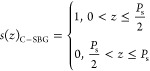
3

Here, the + first channel (*m* = +1) is used as the filter’s working channel. By calculating the Fourier coefficients, the coupling coefficient κ of the + first subgrating in the C-SBG is 1/π times that of the uniform Bragg grating (UBG). It is important to note that a lower κ results in a narrower stopband width and a weaker extinction ratio (ER), which for filters translates to a narrower tuning range and a lower SMSR. The current mainstream solution to address the low ER of C-SBG is to compensate for the insufficient κ by designing a longer cavity length. However, in SOI-based single-mode waveguides, increasing the cavity length results in more scattering, which leads to higher propagation losses. Additionally, this approach is not conducive to the miniaturization and integration of photonic chips. To address the issues associated with C-SBG, we implemented a 4PS-SBG structure in our design. In comparison, the 4PS-SBG structure divides each *P*_s_ into four equal segments, with each adjacent segment having a π/2 phase shift (PS). The new corresponding *s*(*z*) is as follows^28^
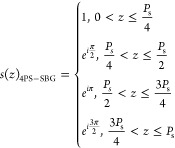
4

**Figure 1:**
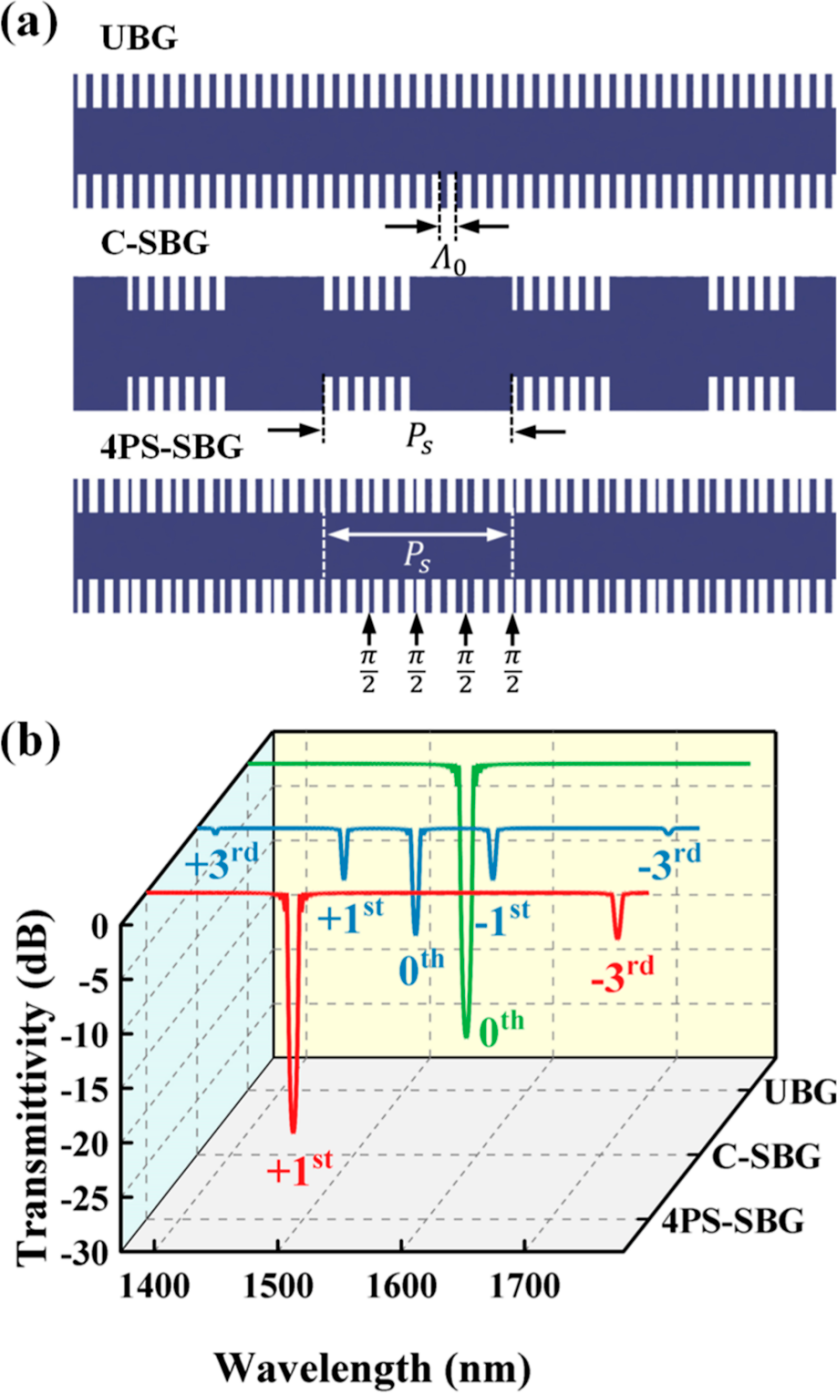
(a) Schematic and (b) transmission spectrum of the UBG, C-SBG, and 4PS-SBG.

Similarly, by calculating Fourier coefficients, we found that the 4PS-SBG structure results in a κ value for its + first subgrating channel that is approximately 0.9 times that of a UBG. This means that when using the + first passband as the working channel for the filter, the 4PS-SBG structure allows for a shorter cavity length than the C-SBG structure while achieving the same ER performance and a wider stopband. Figure 1a,b, respectively, shows the schematic and the transmission spectra of the UBG, C-SBG, and 4PS-SBG, calculated using the transfer matrix method (TMM).^29,30^ Notably, the 4PS sampling structure not only enhances the + first subgrating channel by approximately three times but also effectively suppresses the zeroth order channel, preventing crosstalk from spurious signals in the zeroth order channel to the microwave signal, compared to the C-SBG.

**Figure 2:**
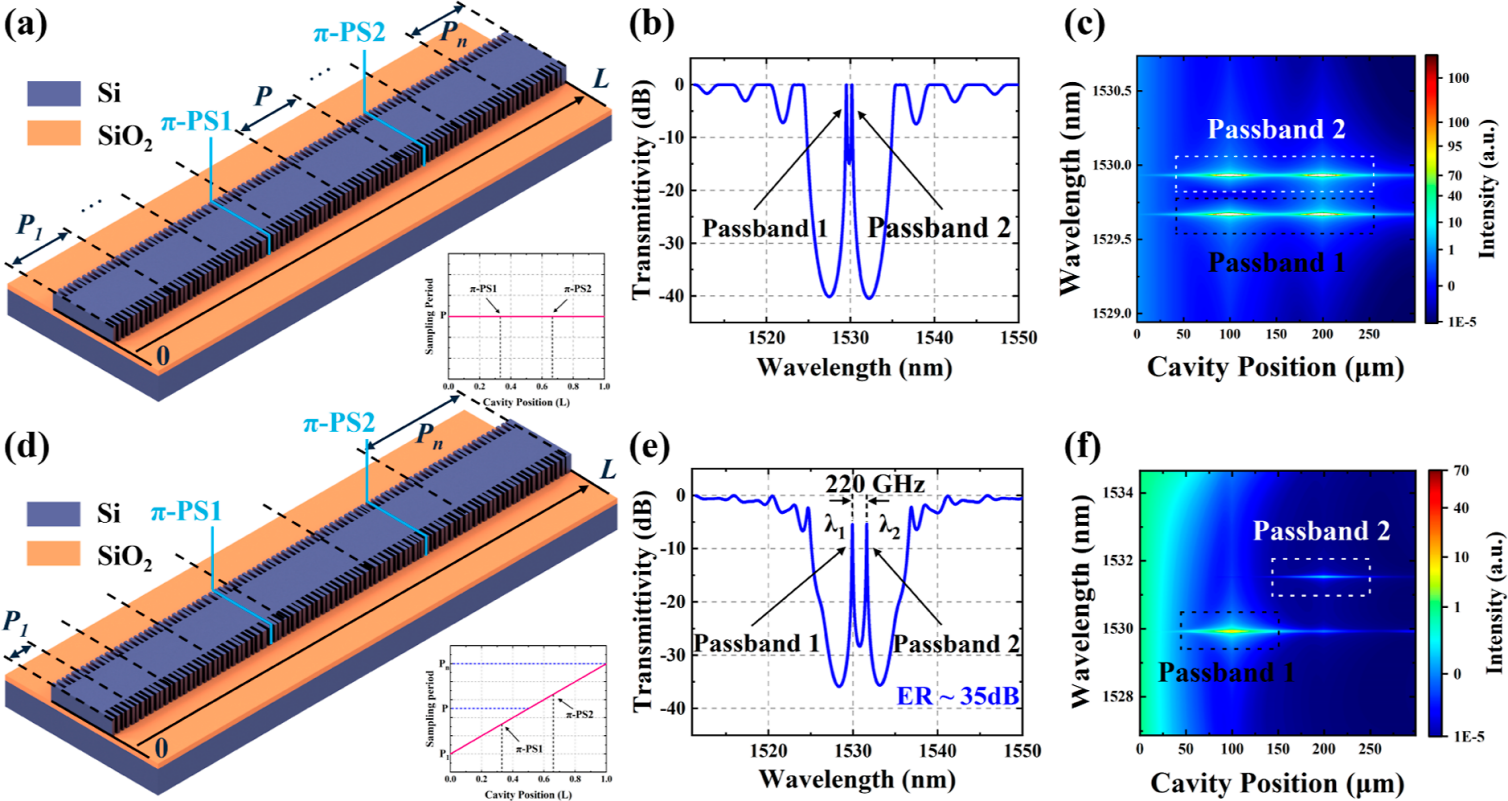
(a) Schematic representation of the uniform 4PS-SBG and sampling period distribution. (b) Transmission spectrum of the uniform 4PS-SBG. (c) Photon distribution along the cavity of the uniform 4PS-SBG. (d) Schematic representation of the chirped 4PS-SBG and sampling period distribution. (e) Transmission spectrum of the chirped 4PS-SBG. (f) Photon distribution along the cavity of the chirped 4PS-SBG.

Typically, introducing two π-PS points in a C-SBG-based cavity can generate two transmission peaks in all subchannels. Similarly, adding more PSs would create multiple passbands within the stopband. However, maintaining uniform frequency spacing and consistent peak intensity across the passbands becomes increasingly challenging. Furthermore, in the case of microwave signals generated by beating the filtered light, multichannel configurations are more prone to producing harmonic signals in the spectrum beyond the desired RF signal than dual-channel configurations. Figure 2a illustrates a schematic of a uniform 4PS-SBG with two π-PSs. To generate two distinct passbands, two π phase shifters (π-PS1 and π-PS2) are strategically placed at one-third and two-thirds of the total cavity length (*L*). Here, *L* is 300 μm, the seed grating period Λ_0_ is 351 nm, and the sampling period *P*_S_ is 3.113 μm, which places the + first channel at 1530 nm. The transmission spectrum and photon distribution of the entire grating are crucial for predicting the filter’s performance. Therefore, we used the TMM to obtain the transmission spectrum and the corresponding photon distribution of the uniform 4PS-SBG, as shown in Figure 2b. In the transmission stopband, two distinct passbands (referred to as passband 1 and passband 2) can be clearly observed, which are introduced by PS1 and PS2. Similarly, the photon distribution can also be calculated using the TMM. Figure 2c shows the distribution of photons along the cavity length within the stopband wavelength range. The photon distributions of passbands 1 and 2 nearly overlap, indicating that the formation of these passbands results from the combined accumulation of incident light at the two PS points, with the light then transmitted through the other end of the grating. This overlap causes the optical fields of both passbands to change simultaneously when the PS magnitude at either PS point is adjusted, making independent tuning of the passbands impossible. This behavior arises from the distributed feedback nature of the uniform grating, where no fixed reflection points exist at specific locations.

To address the aforementioned issue, we introduce an equivalent chirped grating design (Figure 2d), which ensures that light of a specific frequency is reflected by the specific sections of the chirped grating.^31^ By introducing a linear chirp to the sampled grating, the sampling periods at the two PS locations differ, leading the two passbands to originate from different optical cavities, which enables independent tuning of the passbands. The linear chirp sampling period distribution satisfies the following equation
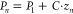
5where *P*_1_ is the first sampling period and *P*_*n*_ represents the *n*th sampling period at the start position of *z*_*n*_. *C* refers to the chirp rate, defined as the ratio of the difference between the maximum and minimum sampling periods (*P*_*n*_ – *P*_1_) to the cavity length *L*. In our device, the chirp rate, *C*, is set to 200 nm/mm. Due to the introduction of the chirped structure, the sampling periods corresponding to the two π-PSs are different. Each PS introduces a transmission peak at a wavelength that satisfies the local Bragg condition. Figure 2e is the transmission spectrum of the chirped 4PS-SBG calculated using the TMM. Two narrow passbands, referred to as passband 1 and passband 2 (respective center wavelengths are λ_1_ and λ_2_), are clearly observed within the transmission stopband, aligning with the phase discontinuities caused by PS1 and PS2, respectively. The initial frequency spacing between the dual passbands within the stopband is 220 GHz. Increasing the chirp rate or reducing the PS spacing can expand the frequency spacing, thereby reducing the spectral overlap in the initial spectrum. However, a spacing of 220 GHz represents an optimal trade-off between the tunable range of the passbands, filtering resolution, and photonic isolation between the dual passbands. The spatial photon distribution of the two passbands is shown in Figure 2f. Compared to uniform sampled Bragg gratings, the optical fields of passbands 1 and 2 are spatially concentrated around π-PS1 and π-PS2, respectively, with their photon distributions clearly separated. This separation indicates the mutual independence of the two passbands. In the stopband, longer wavelengths are reflected nearer the end of the grating, where the grating period is longer, while shorter wavelengths are reflected nearer the beginning of the grating, where the period is shorter. It is worth noting that the introduction of chirping causes the reflection positions of the two passbands within the grating to differ. As a result, less optical power reaches PS2, which is farther from the input port, than the power passing through PS1. This variation in power transmission leads to differences in propagation delays within the grating, causing dispersion and a reduction in the peak intensity of passband 2.

**Figure 3:**
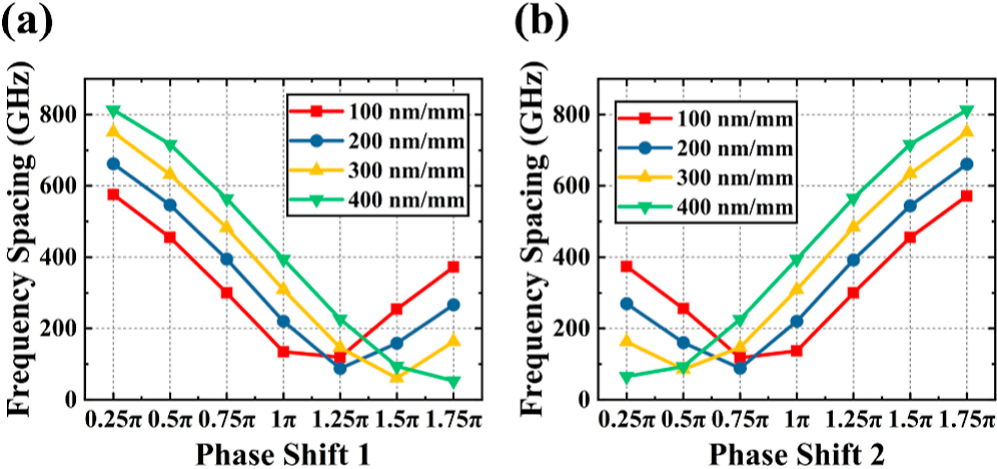
Calculated frequency spacings at different chirp rates as a function of the amplitude of PS1 (a) and PS2 (b).

Therefore, when we modulate the value of a specific PS, only one corresponding wavelength is adjusted, while the other remains unchanged. In Figure 3a, we calculate the frequency spacing as a function of the values of PS1, with PS2 fixed at π, under various chirp rates. The results show that increasing only the PS of PS1 leads to a red shift of λ_1_, while the corresponding λ_2_ of PS2 remains constant, leading to a decrease in the frequency spacing. Similarly, in Figure 3b, when only the PS of PS2 is increased, λ_1_ remains unchanged, while λ_2_ is shifted away from λ_1_, increasing the frequency spacing. This method achieves modulation of the filter passband spacing. A higher chirp rate results in a wider separation between the dual passbands. Our calculations show that, at a chirp rate of 200 nm/mm, the dual passband separation frequency can be tuned by over 600 GHz, marking a significant improvement compared to previous similar studies.^5,14,15^

For details on the heating distribution and thermal crosstalk of the 4PS-SBG PF, please refer to Supporting Information, Part B. This section includes simulations of the thermal distribution and thermo-optic effects of the MH performed using COMSOL. The thermal effects on adjacent phase shifters beyond 100 μm are negligible.

## Fabrication and Characterization

**Figure 4:**
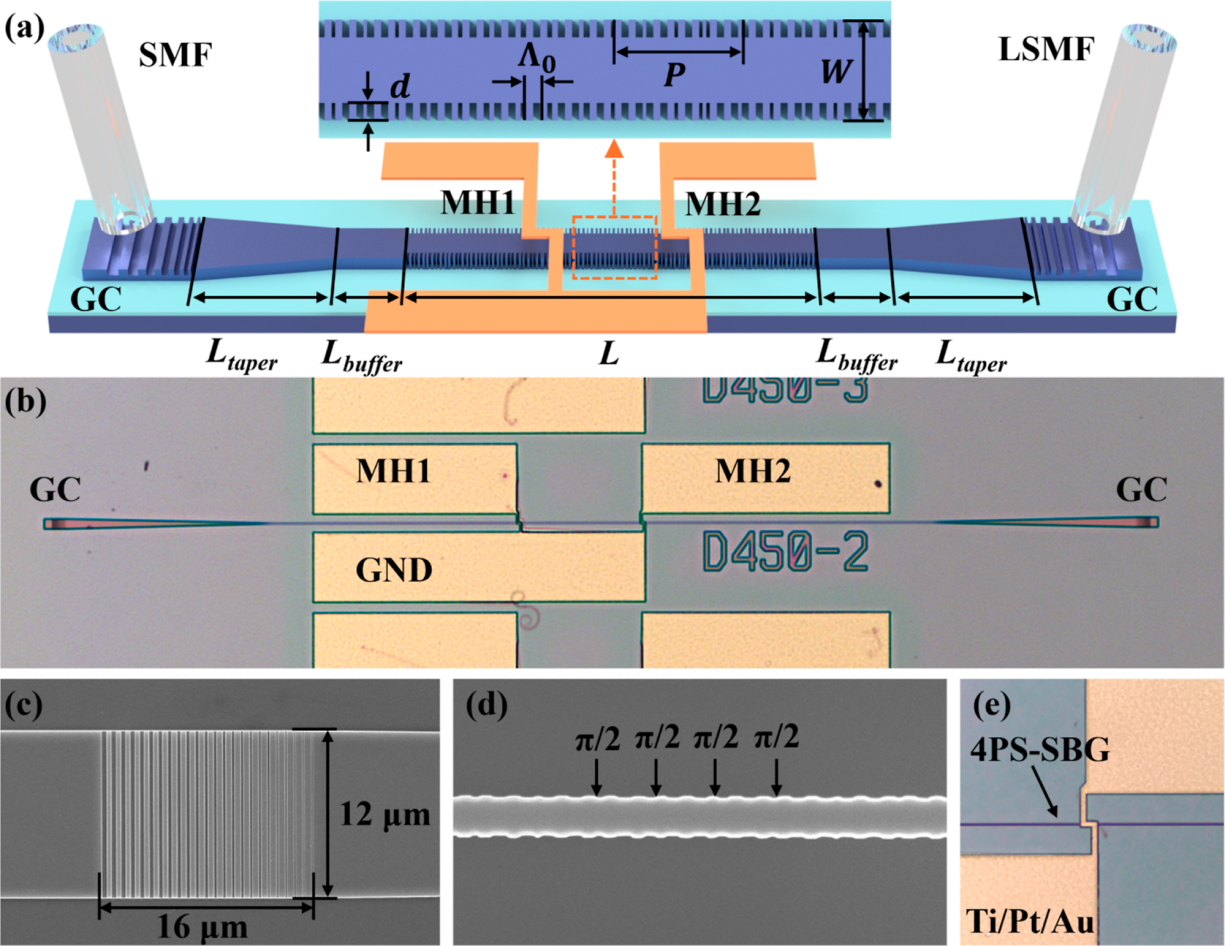
(a) Schematic representation of the PF with the GC, 4PS-SBG, and MHs. (b) Optical microscopy images of the device after fabrication and SEM images of the GC (c), SEM image of the 4PS-SBG (d), and optical microscopy image of the resistor wire of the MH (e).

Figure 4a shows a schematic of the proposed device, where the 4PS-SBG section (*L* = 300 μm) is at the center of the device and the two tapers (*L*_taper_ = 250 μm) for connecting the 4PS-SBG to the grating couplers (GCs, 12 μm wide and 16 μm long) are symmetrically integrated on both sides. Buffer waveguides, each 10 μm long (*L*_buffer_), are placed on both sides to connect the taper and the GC with varying waveguide widths, ensuring a smooth optical mode transition within the waveguide. The SOI wafer has a 220 nm top silicon layer and a 2 μm buried oxide (BOX) layer on a 675 μm thick silicon substrate. Two π-PSs are strategically embedded at 1/3 *L* and 2/3 *L* positions along the 4PS-SBG cavity. As depicted in the zoomed-in view, the side-wall gratings of the 4PS-SBG are symmetrically arranged, featuring a seed grating period (Λ_0_) of 351 nm, a grating recess (*d*) of 25 nm, and a ridge waveguide width (*W*) of 520 nm. The chirped 4PS-SBG waveguide has an effective refractive index of 2.47 at the wavelength of 1535 nm. The grating samplings adhere to eq 5. The device comprises 96 complete sampling units distributed throughout its cavity, denoted by *n* = 96. The chirp rate *C* is set at 200 nm/mm, with the first sampling period *P*_1_ of 3.083 μm, aligning the + first order channel near the wavelength of 1530 nm. The 60 nm difference in sampling periods between *P*_1_ and *P*_*n*_ can be precisely achieved by using electron-beam lithography (EBL), which offers a resolution of 0.5 nm.

The device fabrication process follows the standard procedure used for commercial 220 nm SOI wafers. For more details, please refer to Supporting Information, Part C.

The top-view optical microscopy image of the fabricated filter device is depicted in Figure 4b. Figure 4c presents a zoomed-in SEM image of the fabricated GC, while Figure 4d showcases the fabricated 4PS-SBG structure. Additionally, Figure 4e illustrates the MHs fabricated on the surface of the cladding and its relative position to the embedded grating waveguide. The MH consists of contact pads and heating resistance wires, with serpentine heating wires positioned directly above the two PS regions, each with a width of 2 μm and a resistance of 8.9 Ω at 20 °C.

**Figure 5:**
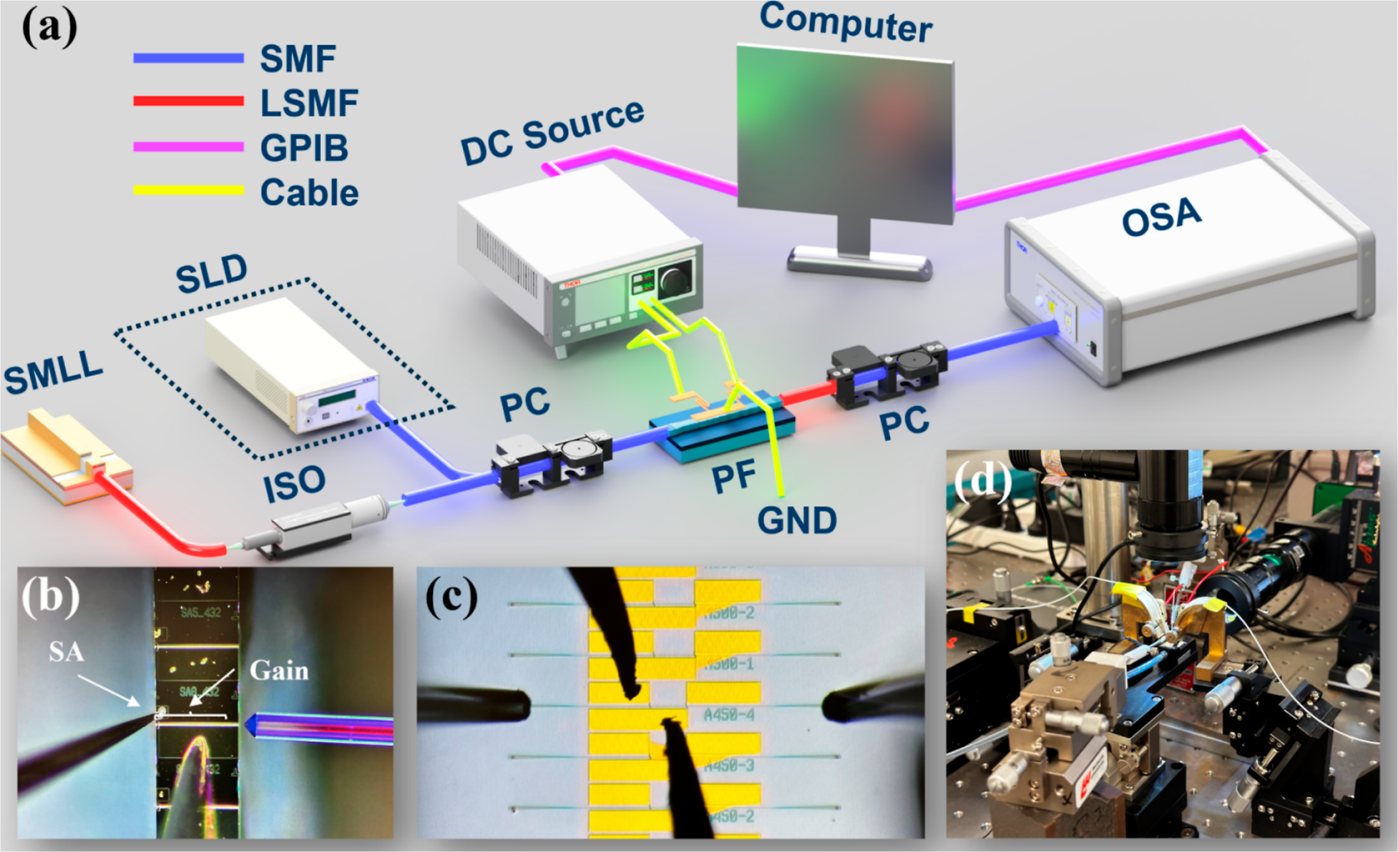
(a) Schematic diagram of the characterization setup, (b) light source coupled from the SMLL using a lensed fiber, (c) microscopy image of the PF monolithically integrated with GCs, showing metal probes connected to MHs and cleaved SMFs connected to the GCs, and (d) photograph of the measurement stage.

In Figure 5a, the experimental setup for device characterization at room temperature includes a superluminescent diode (SLD) as the light source, with a center wavelength of 1551 nm and a 3-dB bandwidth of 30 nm, as well as a 100 GHz frequency spacing SMLL with a center wavelength of 1535 nm. A single-mode fiber (SMF) is connected to the SLD, and a polarization controller directs the light toward the input GC surface. At the output end, a lensed single-mode fiber (LSMF) with a coating is used to couple the output light from the GC surface, minimizing unwanted resonances from a Fabry–Pérot (FP) cavity formed between the fiber and the GC. The input and output SMFs are positioned directly above the GCs, with a vertical offset angle of 15°. The output optical signal is observed using an optical spectrum analyzer (OSA) with a resolution bandwidth (RBW) of 0.06 nm. A polarization controller (PC) is placed before the OSA to ensure that only the TE mode light is characterized. The output wavelength of the device can be adjusted by varying the injection current applied to the MH contact pads (*I*_MH_).

It is worth noting that during the continuous tuning experiments of the filter, only the SLD was used as the light source. For subsequent OFD applications, a 100 GHz SMLL is used as the input source (see Figure 5b) When the SMLD is used as the input source, an optical isolator (ISO) is also introduced to prevent reflected light from the PF from damaging the SMLL.

To demonstrate the filtering properties and associated parameters, we applied varying currents to the MH pads and measured the resulting resonant peak shifts in the transmission spectrum, as shown in Figure 5c. An automated measurement system was designed and implemented using a general–purpose interface bus (GPIB) to interface seamlessly with the measurement instruments. Controlled via LABVIEW software, the system enabled efficient and rapid data acquisition. This setup not only facilitates high-throughput measurements but also ensures consistent precision across multiple experimental runs. Figure 5d shows a photograph of the measurement stage in our test lab.

## Results and Discussion

### Integrated Photonics Filter Characterization

**Figure 6:**
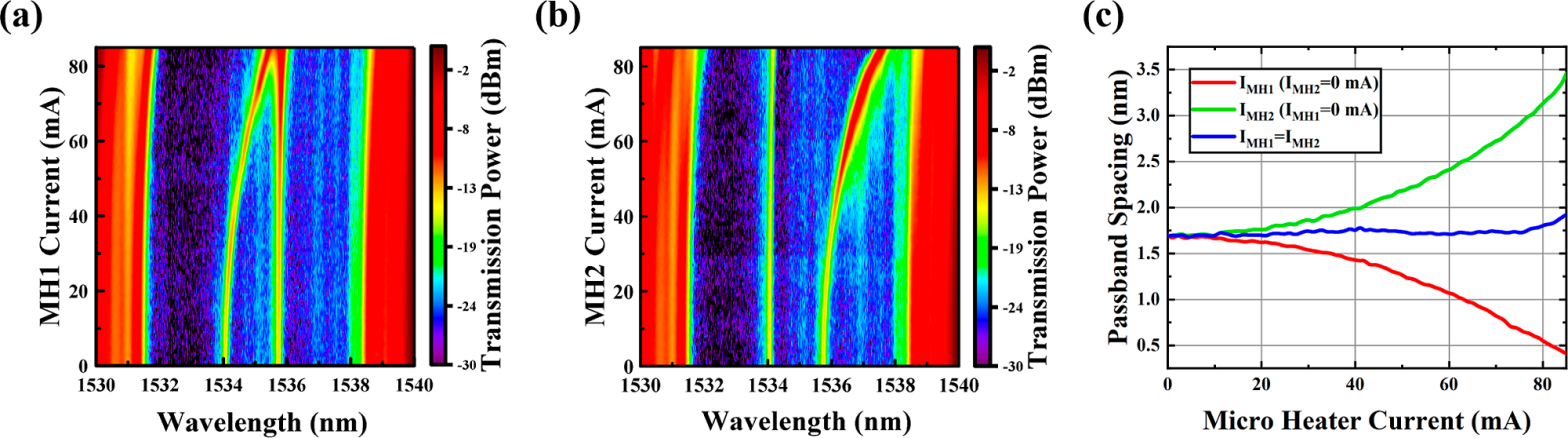
(a) 2D optical spectra with only MH1 modulation; (b) 2D optical spectra with only MH2 modulation; and (c) wavelength gap of the two passbands versus MH currents.

Figure 6a shows the transmission spectrum when only *I*_*MH*1_ is tuned from 0 mA to 85 mA, in which when the modulation current is 0 mA, the spectrum reveals dual passbands (*Q* factor: 1.18 × 10^4^) located within a 7.5 nm range, centered at the 1534.9 nm stopband. The frequency separation is 216.3 GHz, closely matching the simulation results and demonstrating a balanced and consistent intensity distribution. The 4.1 nm red shift of the center wavelength compared to the simulation result may be attributed to etching the ridge waveguide to a height of 210 nm instead of 220 nm as in the simulation. This reduction in height increases the effective index, which, in turn, shifts the center wavelength. The relatively low *Q* factor compared to the simulation may be due to the inadequate RBW of the OSA and fabrication imperfections, such as sidewall and surface roughness of the ridge waveguide, which introduce additional scattering losses and, thus, reduce the *Q* factor. The effective grating coupling coefficient κ for the + first channel is measured to be 241/cm, and the measured propagation loss of the designed 4PS-SBG waveguide is 10.9 dB/cm at a wavelength of 1550 nm.

Figure 6a also illustrates that during the scan of *I*_*MH*1_ from 0 to 85 mA, the thermo-optic effect of the PS induces a red shift of passband 1. The center wavelength of passband 2 remains unchanged during MH1 modulation, allowing for independent tuning of a single band. The separation frequency changes from 216.3 to 52.1 GHz. The consistent positioning of the stopband suggests precise alignment and geometric layout of the MHs, with minimal residual heat leakage. Similarly, as depicted in Figure 6b, independent modulation of passband 2 can also be achieved by solely tuning *I*_MH2_. When *I*_MH2_ is tuned from 0 to 85 mA and *I*_MH1_ = 0 mA, the separation frequency changes from 216.3 to 439.5 GHz. Furthermore, as shown in Figure 6c, analysis of the changes in passband spacing during MH modulation from 0 to 85 mA reveals a continuous modulation range from 52.1 to 439.5 GHz, enabling dynamic tuning of passband separation. According to the simulation results in Figures 6a and 4b, the tuned wavelength gap can be up to 661 GHz if the MH wire is optimized to prevent breakage when a high injection current (>85 mA) is applied. Experiments on synchronous modulation of the dual passbands by tuning *I*_MH1_ and *I*_MH2_ from 0 to 85 mA simultaneously were also conducted, maintaining a relatively stable wavelength spacing and further confirming the independent tuning characteristic of the dual passbands.

### Optical Frequency Division Experiments

**Figure 7:**
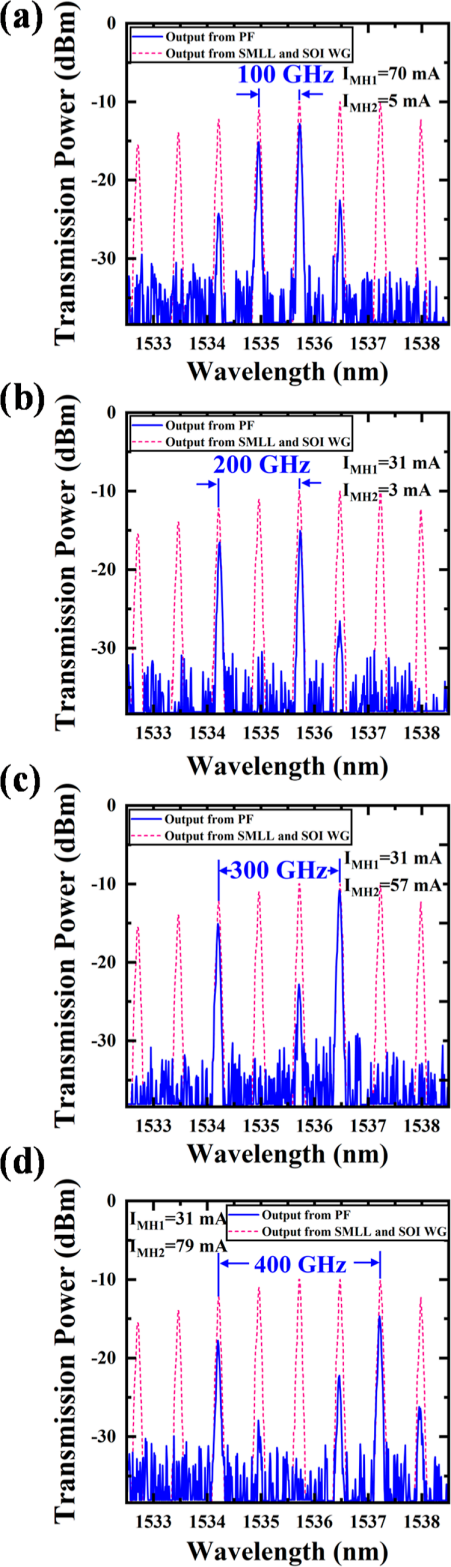
Output spectrum of the OFC signal from the SMLL and SOI ridge waveguide (dashed line) and the OFC filtered by the PF with spacings of (a) 100 GHz, (b) 200 GHz, (c) 300 GHz, and (d) 400 GHz (solid line).

For the OFD experiments, a passive SMLL is designed and fabricated using the AlGaInAs/InP material system to produce an optical frequency comb (OFC) with a 100 GHz spacing. For detailed parameters and performance of the SMLL used as the input source, please refer to Supporting Information, Part D. The isolated laser optical signal, after passing through the ISO, was coupled into the PF. A direct current (DC) electrical signal from a current source controller was applied to the MH via contact pads. The optical signal output from the PF was then transmitted to an OSA after the polarization direction was adjusted by the PC. The output spectra shown in Figure 7a–d were obtained by modulating the driving currents of MH1 and MH2, as depicted in the respective figures. The blue solid line represents the output optical spectrum after the PF, while the pink dashed line indicates the reference output spectrum of the SMLL after passing through a ridge waveguide of the same length as the filter. An analysis of the transmission characteristics of the dual wavelength PF revealed that the output spectra closely followed the trends observed using the SLD source, as shown in Figure 6c. Specifically, when the current applied to MH1 was increased from a low to high value, passband 1 shifted toward passband 2, reducing the separation frequency of the PF. In contrast, modulation of MH2 caused passband 2 to shift away from passband 1, increasing the separation frequency. Figure 7a–d illustrates four distinct filtering configurations with separation frequencies of 100, 200, 300, and 400 GHz, demonstrating a tunable frequency division range from 100 to 400 GHz. These configurations correspond to the modulation currents *I*_MH1_ and *I*_MH2_ shown in the figures. In Figure 7a, side modes appear near the desired main mode. This occurs because, under the 100 GHz filtering configuration, passband 1 shifts closer to passband 2 and partially overlaps, reducing the Q factor of the dual passbands. As a result, some side modes leak into the main filtering window. A similar effect is observed in Figure 7d, where passband 2, modulated closer to the sidelobes, exhibits an increased bandwidth, reducing the suppression of edge modes near the main mode. The most straightforward solution to mitigate these issues is to increase the grating coupling coefficient, thereby improving the passband resolution. When the PF is tuned to select the 200 GHz optical frequency spacing (Figure 7b), the maximum SMSR exceeds 10 dB. The minimum PF insertion loss measured is 0.85 dB at a wavelength of 1536.5 nm.

## Conclusions

In summary, we have proposed a dual-frequency independently tunable PF integrated on an SOI platform. Both simulation and experimental results demonstrate that the introduction of the equivalent chirp enables independent photon distribution in the dual passbands with minimal thermal crosstalk from the metal MHs, allowing for independent modulation of the passbands. The adoption of the 4PS-SBG structure enhances the ER and effectively suppresses the unwanted side modes. The filter exhibits continuous frequency tuning capabilities from 52.1 to 439.5 GHz, with an unprecedentedly wide tuning range of 387 GHz and a narrow passband featuring a −3 dB spectral resolution of 16 GHz. Using an SMLL with 100 GHz frequency spacing demonstrated the PF’s OFD capability from 100 to 400 GHz, achieving an SMSR exceeding 10 dB. Our work addresses the challenges of miniaturization and wideband tunability in multifrequency filters on a silicon photonic platform. This technology can also be extended to terahertz (THz) PF applications. With further optimization of the device and integration of lasers, a tunable fully integrated microwave photonics (MWP) system based on grating filters could be realized. This advancement paves the way for a new class of integrated platforms capable of performing a range of on-chip MWP processing functions, including phase shifters, frequency converters, and RF sources.

## Supporting Information Available

Comparison of this work with other similar research (Part A); heating distribution and thermal crosstalk of the 4PS-SBG PF (Part B); fabrication process (Part C); and SMLL used to generate the OFC (Part D) (PDF)
