# Supplementary material for: Widely Tunable Photonic Filter Based on Equivalent Chirped Four-Phase-Shifted Sampled Bragg Gratings
Source: ACS Photonics. 2025 Jan 27;12(2):899–907. doi: 10.1021/acsphotonics.4c01899 (PMC11843717; doi:10.1021/acsphotonics.4c01899)
Supplement: Supplementary file 1 — ph4c01899_si_001.pdf [file ph4c01899_si_001.pdf]

# Supporting Information for

# Widely Tunable Photonic Filter Based on Equivalent Chirped Four-Phase-Shifted Sampled Bragg Gratings

**Simeng Zhu[1*], Bocheng Yuan[1], Mohanad Al-Rubaiee[1], Yiming Sun[1], Yizhe Fan[1], Ahmet Seckin Hezarfen[1], Stephen J. Sweeney[1], John H. Marsh[1], and Lianping Hou[1].**

1. James Watt School of Engineering, University of Glasgow, Glasgow, G12 8QQ, U.K.

* Correspondence: Simeng Zhu (2635935z@student.gla.ac.uk)
James Watt School of Engineering University of Glasgow
Glasgow, G12 8QQ, U.K
Email: 2635935z@student.gla.ac.uk

Number of pages: 6
Number of figures: 2
Number of tables: 1

# CONTENTS

**Support Information Part A: Comparison of this work with other similar research**

**Support Information Part B: Heating distribution and thermal crosstalk of 4PS-SBG PF**

**Support Information Part C: Fabrication process**

**Support Information Part D: Semiconductor mode-locked laser used to generate the OFC**

**References**

## Support Information Part A: Comparison of this work with other similar research

**Table S1.** Comparison between this work with other similar reported research.

| Ref | Number of channels | Number of Resonators | Independ ent tuning | Modulation method | Modulation range | Modulation current/power |
|---|---|---|---|---|---|---|
| Double ring resonator loaded MZI[13] | >2 | 2 | No | Thermo-optic (TO) | Non-tunable | 20 mW/π<br>sum:200 mW |
| Cascaded distributed feedback Bragg grating resonators[14] | 2 | 2 | Yes | TO | 200 GHz | 104.4 mW (arm1)<br>50.4 mW (arm2)<br>sum:154.8mW |
| Stimulated Brillouin scattering based waveguide[19] | 1 | 1 | No | Acousto-optic | 6 GHz | Not reported |
| Equivalent Phase-Shifted Fiber Bragg Grating[5] | 2 | 1 | Yes | Phase-modulation to intensity-modulation (PM-IM) | 7.4 GHz | Not reported |
| Micro ring[22] | >2 | 1 | No | TO | Not reported | Not reported |
| PS-BG on thin-film lithium niobate[23] | 2 | 1 | No | Electro-optic (EO) | 0.9 nm | 3.83 pm/V |
| This work | 2 | 1 | Yes | TO | 387.4 GHz | 85 mA/64 mW |

Notes: All the references in the table are from the manuscript.

## Support Information Part B: Heating distribution and thermal crosstalk of 4PS-SBG PF

Typically, thermo-optic phase shifts can be achieved either by integrating MHs on top of the waveguide or by utilizing the resistive effect of doped waveguides.[1] The former approach involves using a sufficiently thick protective cladding to prevent the metal from affecting the optical propagation modes, thereby minimizing additional optical losses caused by evanescent field. The latter method, however, provides faster response times. Despite this, the integration of metal MHs avoids the need for ion implantation and annealing, simplifying the fabrication process. Consequently, this paper uses metal MHs to achieve thermo-optic phase shifts. The silicon waveguide has a cross-section of 0.5 μm × 0.22 μm, with a buried oxide layer of 2 μm and a top cladding oxide thickness of 1 μm. The heater metal consists of titanium (Ti)/platinum (Pt)/Gold (Au) with a total thickness of 0.23 μm. The width of the heating wire is 2 μm, where Ti serves as an adhesion layer, Pt acts as the primary heating element, and gold provides protection against oxidation and corrosion.

The heating distribution of the chirped 4PS-SBG PF is simulated using COMSOL Multiphysics in a three-dimensional model (Figure S1(a)). The relationship between the phase shift of the waveguide and the temperature change, $\Delta T$, is given by:[2]

$$\nabla\phi = \frac{2\pi}{\lambda} L \left( \frac{\partial n_{eff}}{\partial T} \right) \Delta T \tag{1}$$

Here, $n_{eff}$ represents the effective refractive index, and $L$ is the resonance cavity length. MH1 and MH2 are thin-film heaters placed on top of the cladding oxide, with a 100 μm spacing between the two heating wires, both sharing a common ground plate. In this simulation setup, we apply the electric potential only to MH2 to observe the effects of thermal crosstalk. As shown in Figure S1(a), MH1 exhibits no significant surface temperature change, while MH2 demonstrates highly localized heating. Assuming ideal heat transfer at the phase-shift point, the cross-sectional thermal distributions of MH1 and MH2 also confirm that when only one MH is working, there is no temperature change inside the silicon waveguide below the other MH, while the heat generated by the working MH penetrates highly vertically through the top cladding $SiO_2$ layer to reach the silicon waveguide below it, with little or no horizontal diffusion. Figure S1(b) shows how the phase shift amplitude of the waveguide changes under the two MHs as a function of the tuning current applied to MH2. For a tuning current of 91 mA, which induces a phase change of 1π in PS2, the phase change in PS1 under the same conditions is only 0.012π. If thermal

crosstalk is defined as the ratio of the phase change in the unmodulated waveguide to that in the modulated waveguide,[3] then for this device with the given MH geometric design, the thermal crosstalk is only 1.2%. This indicates that the MH distribution in this device provides excellent thermal isolation, with the thermal effects on adjacent phase shifters beyond 100 μm being negligible.

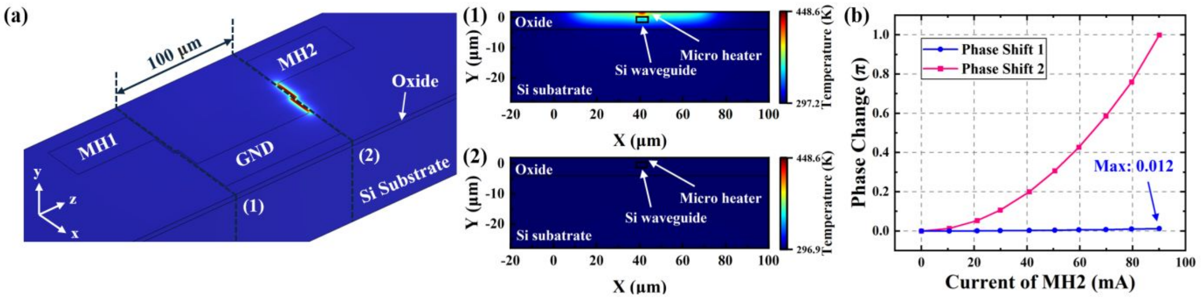


**Figure S1.** (a) Simulated temperature distribution when only MH2 is operating and the corresponding cross sections at positions (1) and (2). (b) Phase change of MH1 and MH2 when injection current is applied only to MH2.

## Support Information Part C: Fabrication process

The fabrication process of the device begins with defining the 210-nanometer-high silicon ridge waveguides and sidewall gratings. This requires the use of hydrogen silsesquioxane (HSQ) resist mask, E-beam lithography (EBL), and inductively coupled plasma (ICP) etching. During etching, an SPTS Rapier DSiE system is used with a gas flow of $C_4F_8$/$SF_6$ at a ratio of 90:30 sccm. Afterward, the HSQ mask is removed using a hydrofluoric acid (HF) solution, leaving a 10 nm residual silicon layer to protect the underlying silicon dioxide. A second round of EBL exposure and dry etching is then performed using polymethyl methacrylate (PMMA, AR-P 642 200 k Anisole 12%, baked at 180°C, and developed in a 2.5:1 MIBK:Isopropyl Alcohol (IPA) solution) as resist and masks. This process forms 110 nm deep GC at both the input and output ends of the 4PS-SBG. Next, to create the top cladding and prepare for subsequent metal deposition, $SiO_2$ deposition and HSQ spin-coating are used to planarize the surface. A 400 nm thick $SiO_2$ layer is deposited on the wafer surface using plasma-enhanced chemical vapor deposition (PECVD), forming the embedded waveguide. Afterward, a 600-nm-thick HSQ layer is spin-coated and annealed at 180°C. Measurements using a Bruker Dektak XT Stylus Profiler showed that the height difference at the waveguide section was reduced from 210 nm to 50 nm, confirming successful surface planarization. Finally, a two-layer PMMA resist (AR-P 642 200k Anisole 15% PMMA and AR-P 679 950k Ethyl Lactate 2% PMMA, baked at 180°C, developed in a 2.5:1 IPA solution) was used in combination with EBL. Metal evaporation was then employed to define the MH components, consisting of 20 nm Ti, 160 nm Pt, and 50 nm Au, followed by lift-off technology.

This surface planarization method is designed to simplify the fabrication process and optimize yield. In typical MH circuits, delamination of the resistive layer is a common primary failure mechanism. This issue arises from excessive interfacial stress caused by a combination of surface roughness and high temperatures. Once the resistive layer delaminates, localized overheating occurs, which further accelerates the delamination process, eventually leading to the burnout of the resistive wire.

In the fabrication process, we systematically optimized the photoresist thickness, electron beam dose, and beam step size (BSS) in the EBL, guided by a series of controlled dose-response and fabrication tests. This careful optimization was crucial for achieving high-resolution sub-wavelength grating periods, smooth sidewall profiles, and precise control over the grating recess.

## Support Information Part D: Semiconductor mode-locked laser used to generate the OFC

For the optical frequency division experiments, a passive SMLL is designed and fabricated using the AlGaInAs/InP material system to produce an optical frequency comb (OFC). It features an asymmetric multiple-quantum-well (MQW) epilayer structure. Figure 5(b) in the manuscript shows an optical microscope picture of the 100 GHz repetition frequency SMLL device. The length of the entire cavity length is 432 μm. The length of the saturable absorption (SA) section is 10 μm, the gain section is 412 μm, and the isolation groove between the gain and SA sections is 10 μm. Its ridge waveguide is 2.5 μm wide. Passive mode locking (ML) is achieved by forward biasing the gain section and applying a reverse voltage to the SA section. To more accurately characterize the optical frequency division performance of the PF, experiments were conducted with the SMLL set to a central wavelength of 1535 nm under pure ML conditions. This was done with a gain current of 100 mA and a reverse bias voltage of -2.3 V on the SA section. The SMLL exhibited a full width at half maximum (FWHM) of 4.5 nm, generating an OFC with a 100 GHz spacing, as shown in Figure S2(a). On its right, shown in Figure S2(b), we provide a zoomed-in view of the localized spectrum. The output optical power from the gain facet was 10 mW. The isolated laser optical signal, after passing through the isolator, was coupled into the PF.

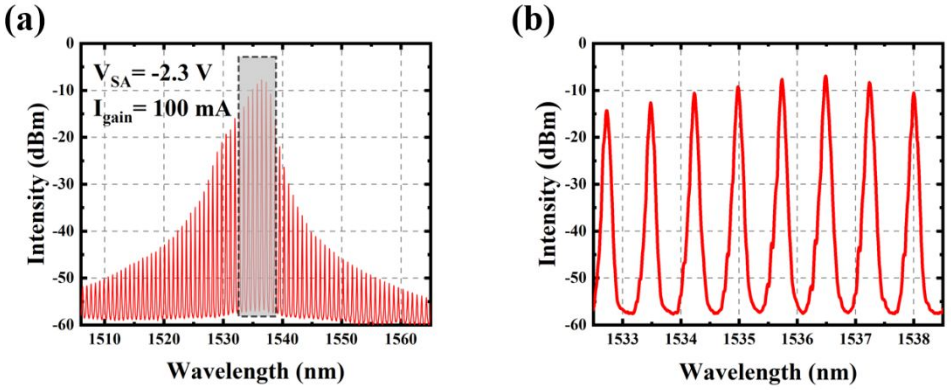


**Figure S2.** (a) Optical spectrum of the gain section facet of the SMLL in the ML state, with a gain section injection current of 100 mA and a reverse bias voltage of -2.3 V applied to the SA section. (b) Zoomed-in optical spectrum of the dotted box labeled in (a).

# References

(1) Watts, M. R.; Zortman, W. A.; Trotter, D. C.; Nielson, G. N.; Luck, D. L.; Young, R. W. Adiabatic Resonant Microrings (ARMs) with Directly Integrated Thermal Microphotonics. OSTI OAI (U.S. Department of Energy Office of Scientific and Technical Information) **2009**, 1–2. https://doi.org/10.1364/cleo.2009.cpdb10.

(2) Cocorullo, G.; Della Corte, F. G.; Rendina, I.; Sarro, P. M. Thermo-Optic Effect Exploitation in Silicon Microstructures. Sensors and Actuators A: Physical **1998**, 71 (1), 19–26. https://doi.org/10.1016/S0924-4247(98)00168-X.

(3) Dong, P.; Qian, W.; Liang, H.; Roshanak Shafiiha; Feng, N.-N.; Feng, D.; Zheng, X.; Krishnamoorthy, A. V.; Asghari, M. Low Power and Compact Reconfigurable Multiplexing Devices Based on Silicon Microring Resonators. OPTICS EXPRESS **2010**, 18 (10), 9852–9852. https://doi.org/10.1364/oe.18.009852.
